# The Importance of Mehran Score to Predict Acute Kidney Injury in Patients with TAVI: A Large Multicenter Cohort Study

**DOI:** 10.3390/jcdd10060228

**Published:** 2023-05-24

**Authors:** Salvatore Arrotti, Fabio Alfredo Sgura, Daniel Enrique Monopoli, Valerio Siena, Giulio Leo, Vernizia Morgante, Paolo Cataldo, Paolo Magnavacchi, Davide Gabbieri, Vincenzo Guiducci, Giorgio Benatti, Luigi Vignali, Giuseppe Boriani, Rosario Rossi

**Affiliations:** 1 Cardiology Division, Department of Biomedical, Metabolic and Neural Sciences, University of Modena and Reggio Emilia, Policlinico di Modena, 41124 Modena, Italy; 2 Cardiology Division, Baggiovara Hospital, 41100 Modena, Italy; 3 Cardiac Surgery Division, Hesperia Hospital, 41125 Modena, Italy; 4 Division of Cardiology, AUSL-IRCCS Reggio Emilia, 42121 Reggio Emilia, Italy; 5 Cardiology Division, Parma University Hospital, 44129 Parma, Italy

**Keywords:** Mehran Score, acute kidney injury, transcatheter aortic valve implantation

## Abstract

Background: Transcatheter aortic valve implantation (TAVI) has developed as an alternative to surgery for symptomatic high-risk patients with aortic stenosis (AS). An important complication of TAVI is acute kidney injury. The purpose of the study was to investigate if the Mehran Score (MS) could be used to predict acute kidney injury (AKI) in TAVI patients. Methods: This is a multicenter, retrospective, observational study including 1180 patients with severe AS. The MS comprised eight clinical and procedural variables: hypotension, congestive heart failure class, glomerular filtration rate, diabetes, age >75 years, anemia, need for intra-aortic balloon pump, and contrast agent volume use. We assessed the sensitivity and specificity of the MS in predicting AKI following TAVI, as well as the predictive value of MS with each AKI-related characteristic. Results: Patients were categorized into four risk groups based on MS: low (≤5), moderate (6–10), high (11–15), and very high (≥16). Post-procedural AKI was observed in 139 patients (11.8%). MS classes had a higher risk of AKI in the multivariate analysis (HR 1.38, 95% CI, 1.43–1.63, *p* < 0.01). The best cutoff for MS to predict the onset of AKI was 13.0 (AUC, 0.62; 95% CI, 0.57–0.67), whereas the best cutoff for eGFR was 42.0 mL/min/1.73 m^2^ (AUC, 0.61; 95% CI, 0.56–0.67). Conclusions: MS was shown to be a predictor of AKI development in TAVI patients.

## 1. Introduction

Severe aortic stenosis (AS) is one of the most common cardiac valvular heart diseases in Western countries. The need for repair—AS is the primary valvopathy which most requires surgery or transcatheter intervention [1]—and the growing prevalence due to the rapid ageing of populations worldwide [2] increases interest on this topic. In the subset of old and often frail patients, transcatheter aortic valve implantation (TAVI) has emerged as an alternative to surgery for those symptomatic and considered unsuitable or at high risk for surgical aortic valve replacement (SAVR) [3]. The major complications for TAVI are related to vascular access, valve deployment, and arrythmias, but also to organ damage such as kidney injury [4]. Unlike percutaneous coronary intervention, where AKI is primarily due to contrast-induced nephropathy (CIN), TAVI patients may develop kidney injury after the procedure due to a variety of factors such as contrast agent administration, concomitant drugs, the need for rapid pacing with resulting hypotension and renal hypoperfusion, blood loss, embolization during the implantation, patient age, the frequent coexistence of atherosclerosis, and postoperative severe inflammatory response syndrome [5]. In order to prevent AKI, it is necessary to be aware of all clinical, anamnestic, and procedural characteristics of the patient, such as older age, previous renal impairment, hemodynamic instability, congestive heart failure class, diabetes mellitus, anemia, and use of large volumes of contrast media [6]. The overall risk for AKI after percutaneous procedures can be expressed by different scores; one of the most used is the Mehran Score (MS), validated in 2004 for percutaneous coronary intervention (PCI) [7]. The predictive value of the MS for development of AKI in the setting of PCI had been widely studied; however, its applicability in TAVI is still not clear and lacks investigation. Therefore, the aim of this study is to evaluate the predictive value of the MS for AKI after the TAVI procedure.

## 2. Materials and Methods

This is an observational, multicenter, retrospective study involving 1180 patients with severe aortic valve stenosis treated with TAVI from 2012 to 2022 in five different Centers: University Hospital of Modena, S.Agostino Estense of Baggiovara, University Hospital of Parma, St. Maria Nuova Hospital of Reggio Emilia and Hesperia Hospital of Modena (North-West area of the Emilia Romagna, a region of the Northern Italy).

The clinical characteristics, including age, gender, body mass index, comorbidities (diabetes, dyslipidemia, hypertension, smoke, and chronic obstructive lung disease), previous events (cancer, stroke, myocardial infarction, coronary artery disease, and atrioventricular block), prior cardiac interventions (coronary artery bypass surgery, PCI, SAVR, and mitral valve replacement), laboratory findings (eGFR and hematocrit), electrocardiographic, echocardiographic, and procedural data of patients were collected in a unique database at the baseline. The study was approved by the local ethics committee.

The indication to perform a TAVI procedure was made after discussion by the local heart team. Patients were classified according to the Society of Thoracic Surgeons (STS) score (low risk <4%, intermediate risk 4–8%, and high risk >8%) and evaluated for percutaneous intervention according to current international recommendations [3].

The access type and the prosthetic valve model balloon, expandable (Sapien XT, Sapien 3, Sapien 3 Ultra, Myval), self-expandable (CoreValve Evolut R, CoreValve Evolut pro, Portico), or other (Direct Flow, Lotus) were chosen by the operators based on echocardiographic and three-dimensional multidetector computed tomography (CT).

The amount of contrast medium was recorded during the procedure. Procedures such as CT and coronary angiography that require the administration of contrast medium were performed from two weeks to 72 h before TAVI. Patients who underwent major surgery, in particular cardiac surgery with CABG, as well as those treated with PCI in the same day of index procedure were excluded. All patients with estimated Glomerular Filtration Rate (eGFR) <60 mL/min/1.73 m^2^, calculated according to the Chronic Kidney Disease Epidemiology Collaboration (CKD-EPI) equation, were treated with our specific “protocol for renal damage prevention”, that was created with a local multidisciplinary team composed by a cardiologist, nephrologist, and an interventional cardiologist, who considered the ejection fraction (EF), eGFR, diabetes, hematologic disease, and peripheral artery disease (PAD). The infusion consisted of sodium bicarbonate, N-acetylcysteine, and NaCl 0.9% solution, in different proportions as follows: (1) patients with preserved EF and eGFR >80 mL/min were treated with 0.9% NaCl 500 mL, (2) patients with severely reduced EF were treated with sodium bicarbonate 20 mEq + N-acetylcysteine 1200 mg in 200 mL of 0.9% NaCl, 1 h before the index procedure followed by N-acetylcysteine 1200 mg after 6 h, (3) patients affected by diabetes or hematologic disease, PAD, and preserved EF were treated with N-acetylcysteine 3600 mg divided in three doses during the day before procedure + sodium bicarbonate 1/6 M 250 mL + 0.9% NaCl 500 mL.

Daily renal function tests were used for monitoring the patients from admission to discharge. Acute kidney injury was defined as an increase in serum creatinine (SCr) by ≥0.3 mg/dL (26.5 mol/L) within 48 h; an increase in SCr to ≥1.5 times baseline, which is known or presumed to have occurred within the prior 7 days; or urine volume < 0.5 mL/kg/h for 6 h [8]. After the procedure, patients were treated with lifelong aspirin (100 mg once daily) and/or clopidogrel (75 mg once daily according to ESC guidelines of the time) [9,10]. Patients for whom oral anticoagulation (OAC) was indicated were treated with lifelong OAC, alone or in addition to aspirin (100 mg once daily), for three months [11].

### Statistical Analysis

Continuous variables were expressed as mean ± standard deviation and compared using analysis of variance (one-way ANOVA) and an independent samples *T* test. Categorical variables were expressed as numbers and percentages and compared using a Chi-square test. Univariate and multivariate Cox regression analyses were used to determine the independent predictors of AKI.

MS included eight clinical and procedural variables: presence of hypotension, congestive heart failure class III/IV by NYHA, eGFR range according to CKD-EPI, diabetes, age >75 years, anemia expressed by the hematocrit value, requirement of intra-aortic balloon pump, and the volume of contrast agent used. As previously introduced, this score was first evaluated for PCI. For this reason, it includes parameters such as the use of IABP and the occurrence of hypotension requiring IABP that are not applicable for a TAVI population, and for which the value of 0 pts was assigned.

Patients were categorized into four risk groups based on MS: low (≤5), moderate (6–10), high (11–15), and very high (≥16) [12].

In the first part of our analysis, we evaluated the sensitivity and the specificity of MS in the prediction of AKI after the TAVI procedure. In addition, we compared the predictive value of MS with each of the AKI-related variables. A receiver operating characteristic (ROC) curve analysis was performed to identify the optimal cutoff point of MS to predict AKI in patients treated with TAVI. The area under the curve (AUC) values were calculated as a measure of test accuracy. Significance for all the analyses considered a two-side *p* value of <0.05. Statistical analyses were realized using IBM SPSS Statistics Software (IBM Corp. Released 2020. IBM SPSS Statistics for Windows, Version 27.0. Armonk, NY, USA: IBM Corp.)

## 3. Results

A total of 1180 patients treated with TAVI were included in the study. The mean age of patients was 82.1 ± 6.7 year. Approximately one-third (27.7%) had a low (≤5) or moderate (6–10) MS. Most patients (73.3%) had a high (11–15) or very high MS (≥16).

The baseline clinical characteristics, echocardiographic, and procedural data of the study population according to the MS classes are summarized in Table 1.

As expected, patients with a very high MS were older, diabetic, with a higher New York Heart Association (NYHA) class, with lower hematocrit and eGFR, and a higher amount of contrast administered. In addition, these patients presented a lower EF, and a significant (moderate–severe) mitral regurgitation.

Access site and device type were not different regarding MS classes. In particular, transfemoral access was used for the majority of patients in all MS classes. The mean hospitalization length, calculated from the intervention day to discharge, was 10.9 ± 5.2 days, higher (12.1 ± 5.5 days) in patients with very high MS (*p* = 0.005).

Post-procedural AKI was observed in 139 patients (11.8%). MS classes and all its variables were individually evaluated in predicting AKI. In the univariate analysis MS classes, the eGFR and NYHA classes were found to be significantly correlated, but only MS classes showed an increased risk of AKI in multivariate analysis (HR 1.38, 95% CI, 1.43–1.63, *p* < 0.01), as shown in Table 2.

**Figure 1:**
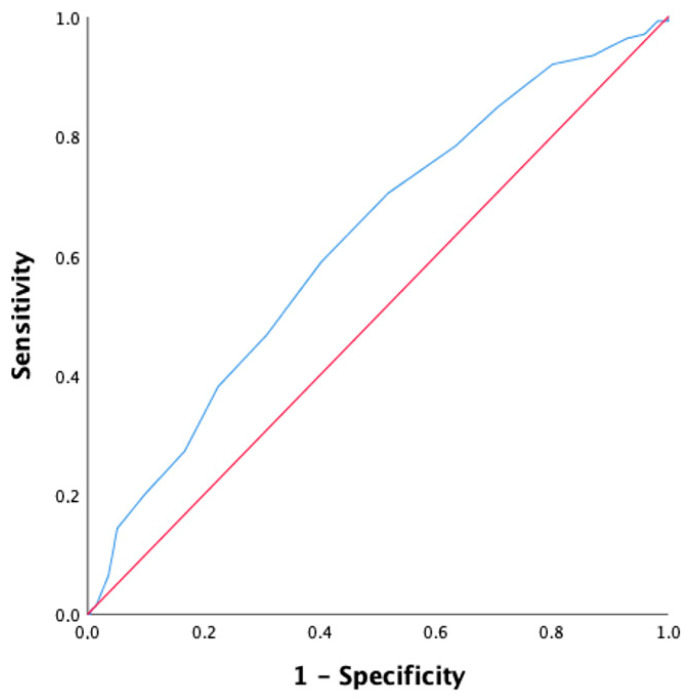
ROC curve for the prediction of AKI using Mehran Score (blue line). AUC for the prediction of AKI was 0.62; 95% CI: 0.57–0.67; sensitivity 70%; specificity 48%. The red line is the reference line.

**Figure 2:**
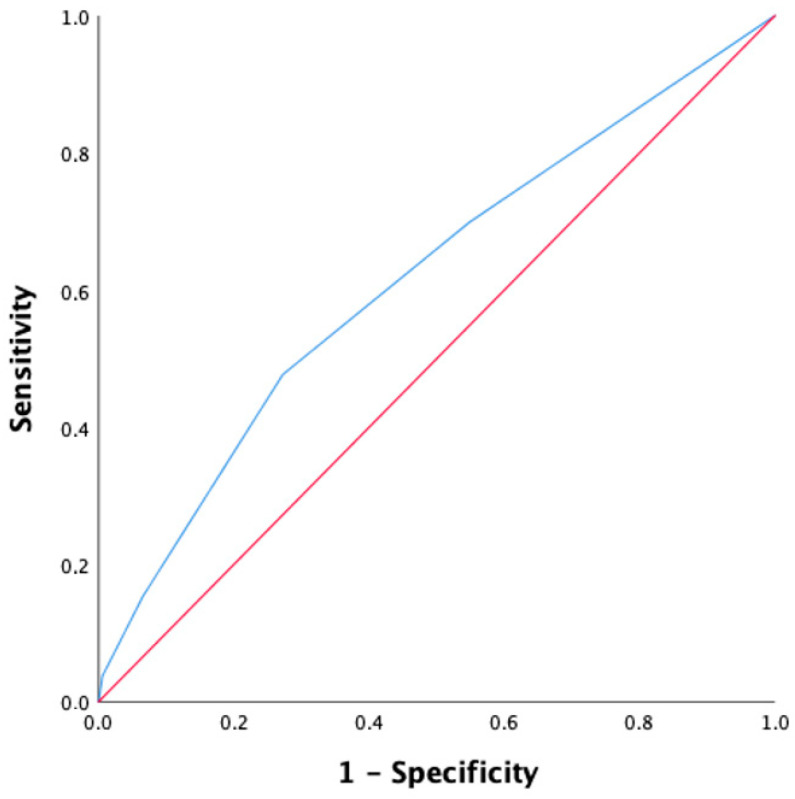
ROC curves for the prediction of AKI using eGFR (blue line). AUC for the prediction of AKI was 0.61;95% C.I: 0.56–0.67; sensitivity 39%; specificity 76%. The red line is the reference line.

The ROC analysis of MS and eGFR revealed that the best cutoff of MS to predict the development of AKI was 13.0 (AUC, 0.62; 95% CI, 0.57–0.67; sensitivity 70%; specificity 48%), while the cutoff for eGFR was 42.0 mL/min/1.73 m^2^ (AUC, 0.61; 95% CI, 0.56–0.67; sensitivity 39%; specificity 76%) (Figure 1 and Figure 2).

The NYHA class was not statistically significant.

Ejection fraction (HR 0.99, CI, 0.96–1.01, *p*-value < 0.45) and moderate-severe mitral regurgitation (HR 0.83, CI, 0.58–1.19, *p*-value < 0.31) were not significantly correlated to AKI.

## 4. Discussion

The present study evaluated the performance of the original Mehran Score (MS), not considering the unsuitable variables described above, in order to predict the development of AKI in patients treated with TAVI.

The incidence of AKI following TAVI varies greatly. AKI incidence in currently available randomized trials of TAVI versus SAVR is relatively low. All patients with AKI in the PARTNER trial were 1.1% (TAVI 4/348, SAVR 4/351); in the PARTNER 2 trial, patients with AKI were 2.2% (TAVI 13/1011, SAVR 31/1021); in the US CoreValve trial, patients with AKI were 10.3% (TAVI 23/390, SAVR 54/357); and in the NOTION trial, patients with AKI were 5.7% (TAVI 1/139, SAVR 9/135) [13,14,15,16]. Data from real-world registries, on the other hand, indicated that AKI might develop in more than 50% of patients, and it is also strongly associated with higher short- and mid-term mortality [17]. Therefore, identifying patients at risk before the procedure could improve the outcomes and reduce renal complications.

MS was developed as a simple risk score of CIN after percutaneous coronary intervention [18]. However, there is no consensus on preventing AKI in patients undergoing TAVI. Some scores developed and validated in different settings, such as WBH, Mehran, CR4EA-TME3AD3, and ACEF scores were tested in the prediction of TAVI-related AKI, but showed limited diagnostic accuracy [19,20].

The main findings of the present observational study were two: (1) in our population, higher Mehran Score was associated with significantly higher risk of AKI (HR 1.36, CI, 1.23–1.63, *p* < 0.001); (2) Mehran score may be used for prediction of AKI after TAVI with a cutoff value of 13, (sensitivity 70 % and specificity 48 % (AUC 0.62, 95% CI, 0.57–0.67)).

Zungur and colleagues have already found that hospital mortality was higher in patients with AKI and was positively correlated with MS. In other words, MS was independently associated with CIN and patients with MS ≥ 13 (sensitivity, 62%; specificity, 68%) were shown to be at high risk for developing AKI. This report was the first to investigate MS in the TAVI setting but was limited by a low number of patients [21]. Notably, the cutoff of MS to predict AKI was the same as ours, despite the different numbers of patients.

Ozdemir et al. showed similar results; the original MS score can predict the development of CIN in patients undergoing TAVI with a cutoff value of 12.5, (61.4% sensitivity and 67.7% specificity) [22]. However, differently from our study, the amount of contrast media was not recorded. In our opinion this was a limitation of the study, as the contrast media is strongly correlated to AKI.

Moreover, AKI in TAVI patients is a combination of the direct nephrotoxic impact of contrast and prerenal azotemia due to pre-, intra-, and postoperative factors such as hypovolemia, hemorrhage, impaired cardiac output, or renal vasoconstriction caused by vasoconstrictive medication [23]. On the other hand, higher age, presence of diabetes, higher NYHA class, lower eGFR, lower hematocrit, and higher amount of contrast medium were associated with higher MS class, as they are all factors of MS.

Estimated Glomerular Filtration Rate (EGFR) evaluated with CKD-EPI, with a cutoff value of 42 mL/min/1.73 m^2^ predicts AKI with a ROC curve slightly inferior to MS (AUC 0.61, specificity 76%, sensitivity 39%). This can be explained by the strong impact that baseline eGFR leads in predicting AKI.

The role of eGFR in development of AKI is well known [24]. Pre-procedural CKD is one of the most frequent comorbidities of TAVI patients and has been found to significantly worsen patients’ prognosis at short and long-term follow-up [25].

The FRANCE-2 Registry assessed the impact of CKD on outcomes following TAVI in 2929 patients. Their findings show that advanced stages of CKD (Stages 4 and 5) are associated with an increase in post-TAVI mortality in both the short and long term [26]. Patients with CKD IV and V, who developed AKI, presented a 9-fold 30-day mortality risk (HR = 9.71, 95% CI, 2.40–39.2, *p* = 0.001) [27].

In conclusion, MS performed better in finding true positives, since it is made up of different variables, while eGFR, as an lone parameter, had higher specificity.

### Study Limitation

Our study had the typical limitations of any retrospective study. Furthermore, intra-aortic balloon pump use and hypotension, two of the parameters considered in the MS, were not present in our population and may be much more rarely present in the TAVI setting than in the coronary one.

The findings of our study should be confirmed by further prospective studies so that the MS can be used in clinical practice for stratifying risk of AKI in the TAVI setting. In addition, it would be interesting to find new parameters specific for the TAVI procedure and its frequent complications that could raise the risk of AKI, such as presence of peripheral artery disease, need for blood transfusion, vascular access complications, or access type.

## 5. Conclusions

MS has been demonstrated as a predictor of AKI development in the TAVI setting, and its use should be diffused in clinical practice in order to decrease the renal complications of TAVI or, at least, better manage their occurrence.

## Figures and Tables

**Table 1:** Clinical, Laboratory, Electrocardiographic, Echocardiographic, and Procedural assessment.

		Low MS *n* = 46	Moderate MS *n* = 281	High MS *n* = 566	Very High MS *n* = 287	*p* Value
**Age and anthropometric factors**						
Age	Years ± SD	76.8 ± 8.2	79.9 ± 9.9	82.8 ± 5.1	83.6 ± 4.1	**<0.001**
Gender	F% (*n*)	60.9 (28)	47.3 (133)	52.7 (298)	54.4 (156)	0.20
BMI	Kg/m^2^ ± SD	27.1 ± 4.6	26.7 ± 4.3	27.0 ± 6.5	26.5 ± 4.8	0.60
**CV Risk factors and Comorbidity**						
Diabetes	%	4.3 (*n* = 2)	8.5 (*n* = 24)	21.6 (*n* = 122)	53.0 (*n* = 152)	**<0.001**
Dyslipidemia	%	63.0 (*n* = 29)	67.4 (*n* = 188)	69.9 (*n* = 395)	70.4 (*n* = 202)	0.66
Hypertension	%	80.4 (*n* = 37)	89.3 (*n* = 251)	87.3 (*n* = 494)	90.2 (*n* = 259)	0.20
Smoker	%	36.4 (*n* = 12)	36.2 (*n* = 84)	31.2 (*n* = 123)	32.6 (*n* = 63)	0.61
NYHA class						**<0.001**
NYHA I	%	9.1 (*n* = 4)	1.9 (*n* = 5)	0.5 (*n* = 3)	0 (*n* = 0)	
NYHA II	%	79.5 (*n* = 35)	41.5 (*n* = 110)	7.5 (*n* = 42)	1.4 (*n* = 4)	
NYHA III	%	9.1 (*n* = 4)	54.7 (*n* = 145)	85.9 (*n* = 480)	87.1 (*n* = 250)	
NYHA IV	%	2.3 (*n* = 1)	1.5 (*n* = 4)	6.1 (*n* = 34)	11.5 (*n* = 33)	
STS score						0.45
Intermediate risk (4–8%)	%	7.7 (*n* = 3)	19.0 (*n* = 40)	19.9 (*n* = 87)	30.4 (*n* = 75)	
High risk (>8%)	%	33.3 (*n* = 13)	25.6 (*n* = 54)	44.6 (*n* = 195)	47.0 (*n* = 117)	
Chronic pulmonary disease	%	8.9 (*n* = 4)	14.6 (*n* = 41)	15.4 (*n* = 87)	16.0 (*n* = 46)	0.65
Cancer History	%	8.7 (*n* = 4)	9.3 (*n* = 26)	10.6 (*n* = 60)	10.9 (*n* = 31)	0.89
Prior Stroke	%	9.1 (*n* = 3)	12.1 (*n* = 28)	9.9 (*n* = 39)	12.9 (*n* = 25)	0.66
Prior myocardial infarction	%	15.2 (*n* = 5)	14.2 (*n* = 33)	13.7 (*n* = 54)	18.0 (*n* = 35)	0.56
Coronary artery disease	%	21.7 (*n* = 10)	33.6 (*n* = 94)	30.7 (*n* = 173)	28.7 (*n* = 82)	0.34
Prior CABG	%	15.2 (*n* = 7)	14.6 (*n* = 41)	10.2 (*n* = 58)	11.1 (*n* = 32)	0.25
Prior PCI	%	10.9 (*n* = 5)	21.0 (*n* = 59)	24.4 (*n* = 138)	22.0 (*n* = 63)	0.16
Prior BAV	%	0 (*n* = 0)	6.5 (*n* = 15)	9.6 (*n* = 38)	11.9 (*n* = 23)	0.06
Prior SAVR	%	15.2 (*n* = 5)	5.2 (*n* = 12)	4.8 (*n* = 19)	4.1 (*n* = 8)	0.06
Prior MVR	%	6.1 (*n* = 2)	2.6 (*n* = 6)	2.3 (*n* = 9)	2.6 (*n* = 5)	0.63
**Laboratory findings**						
eGFR CKD EPI	mL/min ± SD	77.8 ± 15.1	68.3 ± 20.2	57.2 ± 19.2	43.0 ± 20.3	**<0.001**
Hematocrit	%	40.5 ± 7.7	39.2 ± 4.1	37.0 ± 5.3	33.6 ± 4.4	**<0.001**
**ECG characteristics**						
Right bundle brunch block	%	4.3 (*n* = 2)	10.7 (*n* = 30)	6.9 (*n* = 39)	7.7 (*n* = 22)	0.19
Left bundle branch block	%	8.7 (*n* = 4)	7.1 (*n* = 20)	8.8 (*n* = 50)	6.3 (*n* = 18)	0.57
Prior PM implantation	%	8.9 (*n* = 4)	6.8 (*n* = 19)	9.0 (*n* = 51)	10.8 (*n* = 31)	0.40
Atrial fibrillation	%	30.4 (*n* = 14)	27.8 (*n* = 78)	24.2 (*n* = 137)	30.0 (*n* = 86)	0.28
**Echocardiographic parameters**						
Ejection Fraction	%	56.8 ± 6.9	53.6 ± 9.5	53.1 ± 9.2	51.1 ± 10.1	**0.001**
Severe AR	%	17.2 (*n* = 5)	12.9 (*n* = 28)	12.7 (*n* = 48)	15.0 (*n* = 27)	0.80
Severe MR	%	20.0 (*n* = 6)	16.1 (*n* = 35)	17.5 (*n* = 65)	30.9 (*n* = 55)	**0.001**
**Procedural characteristics**						
Access type						0.19
Trans-femoral	%	89.1 (*n* = 41)	86.1 (*n* = 241)	82.8 (*n* = 467)	82.6 (*n* = 237)	
Trans-apical	%	8.7 (*n* = 4)	9.3 (*n* = 26)	11.3 (*n* = 64)	8.4 (*n* = 24)	
Other	%	2.2 (*n* = 1)	4.6 (*n* = 13)	5.9 (*n* = 33)	9.1 (*n* = 26)	
Valve type						0.23
Self expandable	%	37.2 (*n* = 16)	34.2 (*n* = 95)	29.1 (*n* = 164)	34.8 (*n* = 100)	
Balloon expandable	%	62.8 (*n* =27)	65.8 (n = 183)	70.9 (*n* = 399)	65.2 (*n* = 187)	
Amount of Contrast	mL ± SD	142.2 ± 82.4	168.4 ± 82.8	174.1 ± 84.2	209.4 ± 86.7	**<0.001**
Hospitalization Lenght	Days ± SD	8.1 ± 4.4	8.7 ± 7.2	9.4 ± 8.5	12.1 ± 5.5	**0.005**

In bold we highlighted the significant result of our comparisons.

**Table 2:** Cox regression for AKI prediction in the Univariate and Multivariate analysis.

	AKI (Univariate Analysis)			AKI (Multivariate Analysis)		
	HR	95% CI	*p* Value	HR	95% CI	*p* Value
**MEHRAN Classes**	1.64	1.31–2.06	**<0.01**	1.36	1.23–1.63	<0.01
**Age**	1.02	0.99–1.05	0.15			
**Diabetes**	1.26	0.88–1.81	0.20			
**NYHA**	1.59	1.12–2.25	**<0.01**	1.27	0.86–1.88	0.23
**eGFR CKD-EPI**	1.48	1.27–1.74	**<0.01**	1.21	0.91–1.62	0.17
**Hematocrit**	0.96	0.94–1.01	0.06			
**Contrast**	1.00	0.99–1.01	0.37			

In bold we highlighted the significant result of our comparisons.

## Data Availability

Data available on request due to restrictions (e.g., privacy or ethical).

## Abbreviations

TAVI	Transcatheter Aortic Valve Implantation
CV	Cardiovascular
AS	Aortic stenosis
AR	Aortic regurgitation
MR	Mitral regurgitation
AKI	Acute kidney injury
CKD	Chronic kidney disease
BAV	Balloon aortic valvuloplasty
STS	Society of thoracic surgery
EF	Ejection fraction
MS	Mehran Score
CKD-EPI	Chronic Kidney Disease Epidemiology Collaboration
eGFR	Estimated glomerular filtration rate
SAVR	Surgery aortic valve replacement
OAC	Oral anticoagulation
PM	Pacemaker
SR	Sinus rhythm
MACE	Major adverse cardiovascular events
